# Analysing historical events and current management strategies of wildfires in Norway

**DOI:** 10.1038/s41598-025-08760-2

**Published:** 2025-07-10

**Authors:** Warda Rafaqat, Pedro Sanchez, Dag Botnen, Nieves Fernandez-Anez

**Affiliations:** 1 Western Norway University of Applied Sciences, Bjørsonsgate 45, 5535 Haugesund, Norway; 2 Department of Physics and Technology, University of Bergen, Postboks 7803, 5020 Bergen, Norway; 3 Pau Costa Fundation, Mossen Cinto Verdaguer, 42 Baixos A, 08552 Taradell, Spain; 4 Haugaland Brann Og Redning IKS, Diktervegen 8, 5538 Haugesund, Norway

**Keywords:** Natural hazards, Forestry

## Abstract

The behaviour of wildfires and their occurrence are changing worldwide. This change is especially notable in areas where these events were not common and are now gaining strength, such as in Northern Europe. Norway has suffered unexpected periods of dryness and high temperatures, causing a considerable change in the probability of wildfire occurrence. A clear example of this trend was 2018 when unusual weather conditions caused numerous fires to spread nationwide. This changing trend highlights the need to understand and analyse the current situation to mitigate future impacts and losses. This paper examines recent wildfires in Norway by analysing the events that have happened from 2016 to 2023, the period when data is available. While acknowledging that this period may not be extensive enough to predict future patterns, this analysis provides valuable insights into recent trends and occurrences. During this timeframe, Norway experienced an annual average of 1217 wildfires, burning 2019 hectares per year. Wildfires peak in April and May. Southern Norway, particularly the Southeast, experiences more wildfires due to drier conditions and denser populations, while Northern regions have fewer fires. This study also evaluated climatic conditions, highlighting a strong correlation between the Palmer Drought Severity Index (PDSI) anomalies and the severe drying conditions in 2018, along with other climatic factors such as land surface temperature, precipitation, and wind. Additionally, the normative and operational situation is detailed to show the framework around these events. It provides reflections and recommendations to avoid future disasters, emphasizing the need for improved fire safety measures and proactive fire management.

## Introduction

Wildfires are a major threat to forests, communities, and infrastructure worldwide. While they are a natural part of forest ecosystems and play a role in regeneration, their patterns are changing due to shifting climate conditions. The latest reports from the Intergovernmental Panel on Climate Change (IPCC)^1–4^ show that climate change is driving more extreme weather, with hotter, drier conditions in some regions. This has – in combination with several other factors (forestry, recreation, etc.) – led to a rise in wildfires in recent years^5,6^, and their frequency and intensity are expected to grow in the future^7^. Wildfires not only cause widespread damage to people, property, and the environment but also release greenhouse gases, contributing to global warming and creating a cycle that increases the risk of even more fires^8^. This variation in wildfires has led to detrimental effects such as loss of biodiversity, degradation of forest ecosystems, soil erosion, disruption of atmospheric patterns, and exacerbation of global warming through the release of carbon into the atmosphere. Consequently, wildfires have emerged as a pressing environmental and ecological issue^9–11^. Globally, annual carbon emissions from fires are estimated at 3.53 Pg, comprising 25–35% of the total net carbon dioxide emissions^12,13^. In addition to the adverse effects on air quality, water resources, and long-term ecological resilience, wildfires impose significant economic costs, including firefighting expenses, loss of infrastructure, buildings, livestock, wildlife, and productive forests. In extreme cases, they can tragically result in the loss of human lives. In Norway, the 95% rise in forest and grassland fires from 2017 to 2018 led to an additional 1,079.9 million kroner spent on preparedness, averaging 1.06 million kroner per avoidable fire call^14^. The negative impacts of wildfires extend beyond the immediate danger of flames, as they significantly contribute to air pollution, which can have long-lasting effects on human health, including respiratory issues and cardiovascular diseases^15^.

As part of this situation, wildfire occurrences have notably risen in Northern Europe over the past two decades^16^ with more frequent fires shifting to higher altitudes^17^, resulting in forest degradation^18^. It is estimated that up to 90 per cent of wildfires are caused by human activities, while global warming is directly related to their increased abundance and swifter escalation^16^. Indeed, the unprecedented rise in temperatures and dry conditions makes forests more vulnerable to fires, which are likely to escalate to megafires. Historically, wet, cool summers have been frequent in Northern Europe, with a low number of wildland fires occurring and of low intensity. The fire seasons have been primarily identified during Spring, when the average precipitation is lower, and the vegetation is more prone to ignite, and primarily in seasons following dry winters, which has been identified as one of the factors increasing the risk of wildfires. However, this tendency has not been consistent in the last years when some summer seasons have been remarkably dry and hot^19^. 2018 was an exceptionally dry and hot year in Northern Europe, conditions closely linked to the impacts of climate change. An example was the summer of 2018, with a total of 2115 wildland fires in Norway, twice as many as in 2016 and 2017^16^.

The 2018 European drought and heat wave marked a period of exceptionally hot weather that brought about record-breaking temperatures and widespread wildfires across many parts of Europe during the spring and summer of 2018^20,21^. This phenomenon was part of a broader heat wave impacting the Northern hemisphere, partly attributed to a weaker-than-usual jet stream, which allowed hot high-pressure air masses to persist in the same areas. The European Drought Observatory reports that most affected areas experienced drought conditions, particularly in Northern and Central Europe^22^. The World Meteorological Organization has linked the severe heat waves experienced across the northern hemisphere during the summer of 2018 to climate change in Europe, as well as instances of extreme precipitation. Notably, temperatures in Banak in northern Norway soared to 32 °C (90 °F) on 30 July 2018, a remarkably rare occurrence for a region situated north of the Arctic Circle. Additionally, the first half of July witnessed over 40 wildfires. Oslo also saw its hottest summer day in 80 years, reaching a maximum temperature of 34.6 °C (94.3 °F). As stated in the IPCC report, very rare extremes and compound events, such as the 2018 concurrent heatwaves across the Northern Hemisphere, are often linked to significant impacts. The changing climate is already altering the likelihood of such extreme events, including decadal droughts and extreme sea levels, and this trend will continue with future warming. Compound events and concurrent extremes are expected to become more frequent with increasing global temperatures, leading to a higher probability of low-likelihood, high-impact outcomes. Higher warming levels will increase the likelihood of events unprecedented in the observational record^3^.

In the present context, an unadulterated natural wildfire regime may not be present, emphasizing the imperative of understanding the fire regime for space and time^23^. Fire regime encompasses various parameters such as fire type, intensity, severity, size, spatial pattern, and seasonality^24–26^. This is influenced by the interplay of biotic and abiotic factors^27^. The dynamic nature of the fire regime is affected by climatic conditions and human activities^28^. However, wildfires are complex phenomena that are also influenced by other factors such as preventive measures, economic factors, inheritance laws, or even knowledge of fire management. Temporal and spatial characteristics of fire regimes can be computed from wildfire statistical records^29^.

Understanding the impact of wildfires on flora and the environment is needed in the current situation to avoid higher losses when from the events that will take place in the near future. This requires essential information on the spatial and temporal occurrences of fires. However, crucial data such as fire frequency, seasonality, fire regime, hot spots, susceptibility zones, and vegetation affected by wildfires have not been fully determined yet in the case of Norway. To help with this task, the present study aims to document fire patterns in Norway using the wildfire data collected by the Directorate of Civil Protection (DSB) in the database. This database collects the information provided by the fire reports filled by the fire brigades in charge of the management of the incident. These reports contain information such as the burned area, date and time of initiation, and ignition details. Over 8 years (2016–2023), a total of around 9900 wildfires were recorded, affecting an area equivalent to 2461 hectares of the total burned area. In Norway, wildfires affect smaller areas in comparison with others. Between 2016 and 2023, approximately 9793 wildfires were recorded, burning a total area of about 16,157 hectares. In Norway, wildfires typically affect smaller areas compared to other regions. However, 2018 was a remarkable exception, with the highest number of fires and the largest burned area during this period, resulting in significant economic losses. A 95% rise in forest and grassland fires from 2017 to 2018 led to an additional expenditure of 1079.9 million kroner on preparedness, averaging 1.06 million kroner per avoidable fire call^14^. This unprecedented wildfire activity raised critical questions about the underlying factors, particularly the climatic conditions driving such events. It also emphasized the importance of evaluating how policies and regulations evolved or were introduced in response to this extraordinary year.

It is known that various factors such as climatic conditions, topography, vegetation type, and socioeconomic factors significantly influence fire frequencies and intensities. In this study, we focus on trends in fire occurrence, vulnerable counties, specific periods of high fire activity within a season, and the influence of climatic conditions such as land surface temperature (LST), precipitation, and wind on monthly wildfire variations. Additionally, we analyse the effects of PDSI and LST on wildfires and their connection to large wildfire events in 2018.

Furthermore, we analyse counties with higher fire frequencies, larger burn areas, and maximum burn area per fire by region. To assess spatial risk factors for each county, data on the number of fires, total burn area, and burn area per fire were combined to create a composite risk index. This index identifies counties at maximum risk when all three factors are considered equally significant. Recognizing these patterns can help improve wildfire management and mitigation efforts.

Finally, this study examines the current regulations in Norway related to wildfires and the operational implementation of resources for managing these situations. The goal is to provide a comprehensive overview of the current state of wildfire management in Norway, concluding with recommendations to enhance awareness and mitigate the future impacts of wildfires, especially in addressing extreme years like 2018.

## Data and methods

### Target area

**Fig. 1:**
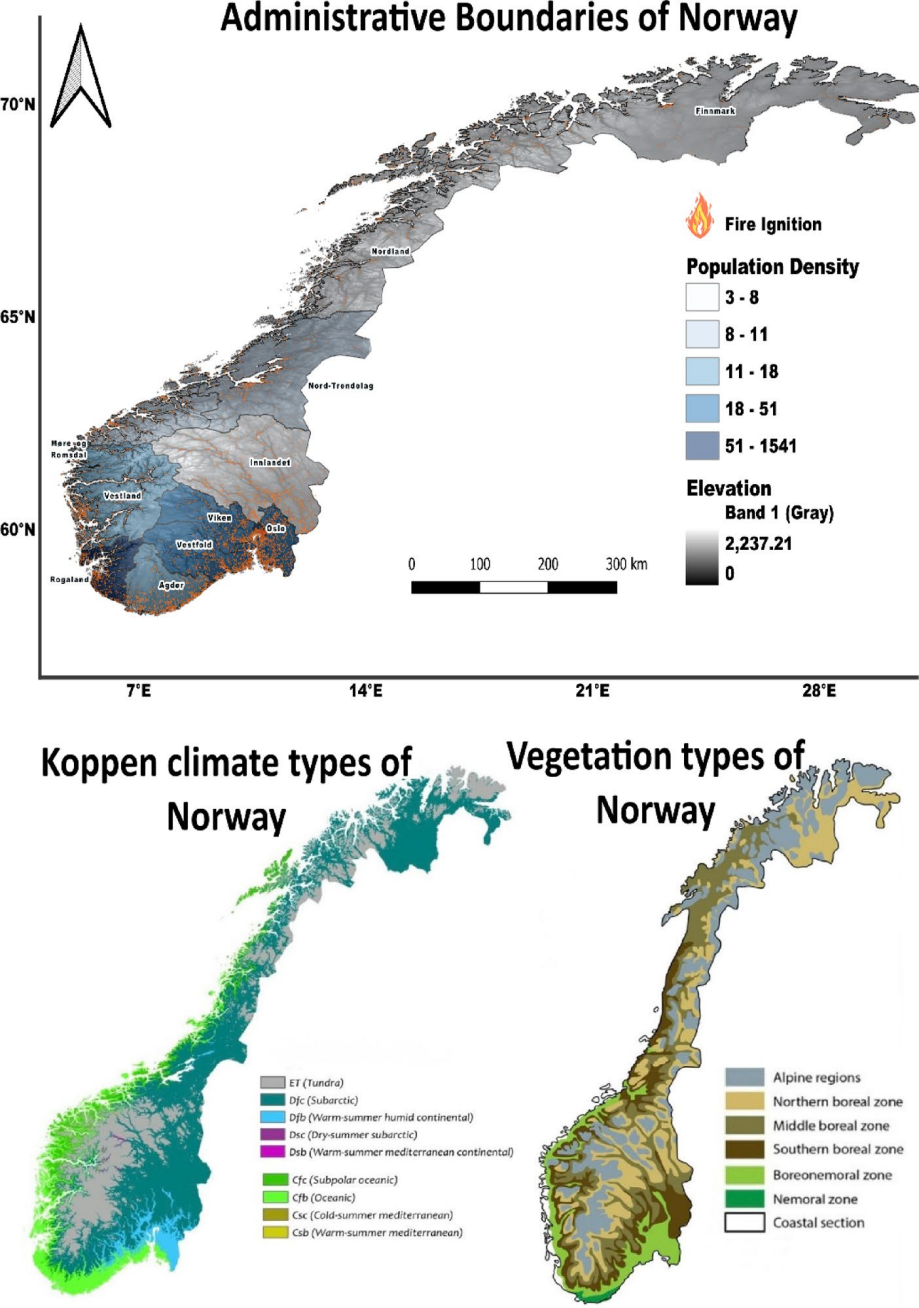
Maps of Norway representing elevation and population density (top), vegetation (bottom right), and Köppen climate types (bottom left). Top map (Elevation and population density): This map was created using QGIS v3.16 (https://qgis.org). Elevation data is sourced from Copernicus DEM (https://spacedata.copernicus.eu/), population density data is sourced from Statistics Norway (SSB) (https://www.ssb.no/en), and fire points data is sourced from DSB fire data (Directorate for Civil Protection) (https://www.dsb.no/en/). Bottom right map (Vegetation): Adapted from Panitz, S., Salzmann, U., Risebrobakken, B., De Schepper, S., & Pound, M.J. (2016). Climate variability and long-term expansion of peatlands in Arctic Norway during the late Pliocene (ODP Site 642, Norwegian Sea). Climate of the Past, 12, 1043–1060. https://doi.org/10.5194/cp-12-1043-2016. Licensed under CC BY 3.0. (https://creativecommons.org/licenses/by/3.0/). Bottom left map (Köppen climate types): Data for Köppen types calculated from WorldClim.org, using a -3°C isotherm to distinguish temperate (**C**) and continental (**D**) climates. Map obtained from Wikimedia Commons (https://commons.wikimedia.org/wiki/File:Norway_K%C3%B6ppen.svg). This file is licensed under the Creative Commons Attribution-Share Alike 4.0 International license (https://creativecommons.org/licenses/by-sa/4.0/).

Here, we analyse data from wildfires that occurred in Norway from 2016 to 2023 (see Fig. 1). Positioned on the Western side of the Scandinavian Peninsula in Northwestern Europe, Norway lies between 57° 58′ N and 71° 11′ N latitude, and 4° 40′ E and 30° 58′ E longitude, placing it centrally within the westerlies and both temperate and polar climate zones^30^. Mainland Norway exhibits a diverse range of physical features, resulting in various climate conditions, encompassing five climate zones according to the Köppen classification^31^. Its proximity to the North Atlantic Current on the western side of the Scandinavian Peninsula moderates the climate to a more temperate level compared to its geographical location. The extensive coastline, numerous fjords, islands, varying altitudes up to 2469 m above sea level, and the transition from maritime to continental climates from West to East contribute to diverse climate patterns on regional and local scales. The Southwestern coastal lowlands of Norway experience relatively temperate climates, while mountainous regions inland often receive substantial precipitation throughout the year, with significant snow accumulation in winter, feeding numerous glaciers. In contrast, the more continental regions of Eastern Norway are notably drier, and the elevated mountain plateaus, particularly in Northern Norway, exhibit subarctic characteristics, including occurrences of permafrost^32^.

Norway spans approximately 38.5 million hectares and is home to just over 5 million inhabitants. The country’s vegetation varies along three gradients: from south to north, from lowlands to mountains, and from coastal areas to inland regions, resulting in seven distinct vegetational regions^33^. On the west coast, characterized by its oceanic climate, heathlands are the dominant vegetation type. Until five decades ago, numerous farmers actively managed these heathlands through cyclical practices, including prescribed fires. However, in the last two decades, nearly all such management activities have ceased, leading to a gradual overgrowth of shrubs and trees across the landscape. This process continues to accelerate today^34^. These heathlands are the main fire risk on the West coast. Conversely, the inland areas, characterized by higher topography, are home to tall trees of various species, such as Scots pine and Norway spruce, resulting in distinct fire behaviour and fire risk compared to other regions. Furthermore, changes in vegetation over the years have altered fire behaviour from pine-dominated forests, prone to fire, to spruce-dominated forests with reduced fire occurrence^1,35^ ^1^.

**Fig. 2:**
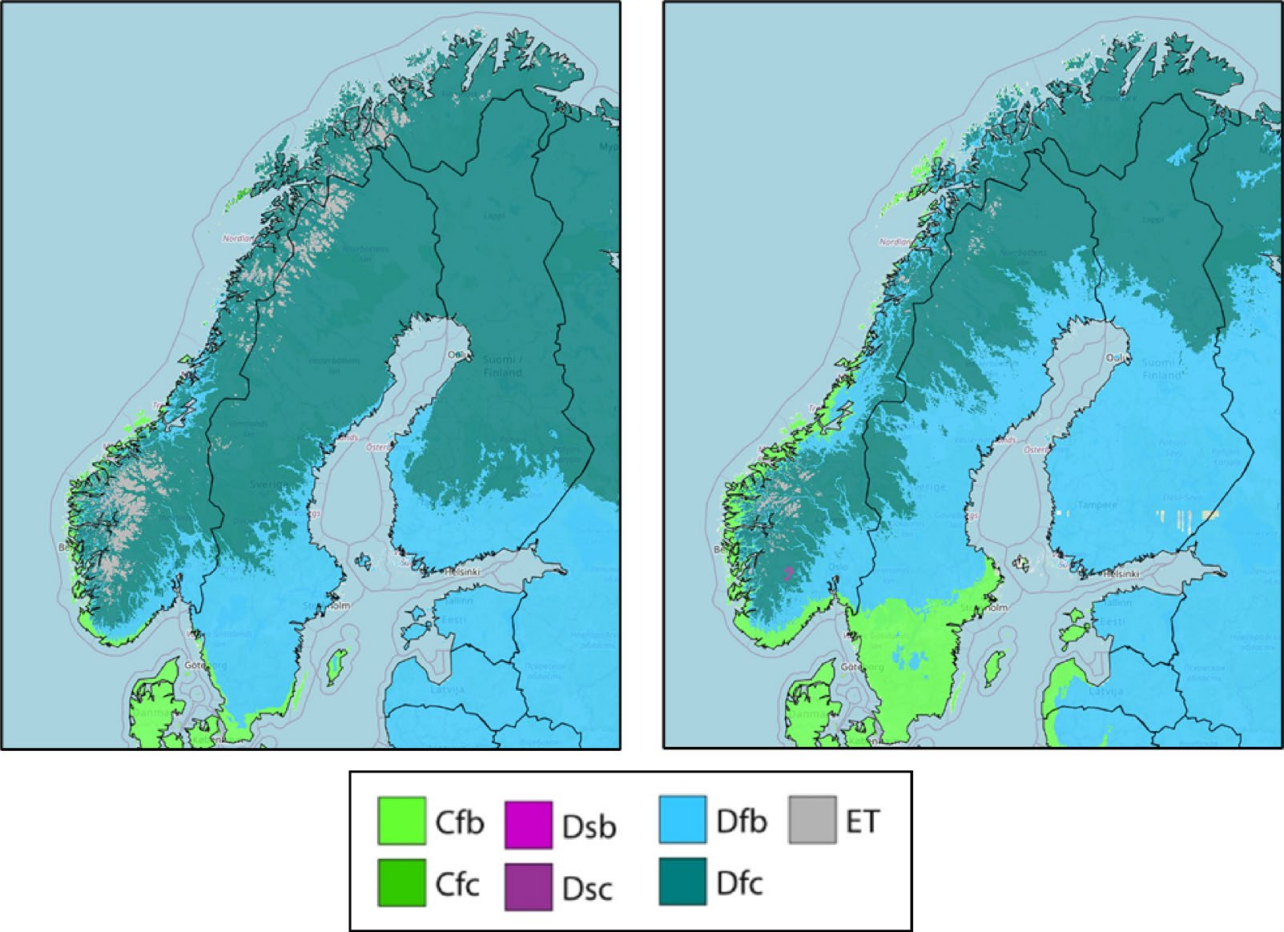
Koppen Geiger evolution in vegetation from the actual one (1991–2020, left) to the last estimation model (2071–2099, right)^38^. Climate classes: Cfb – Temperate, no dry season, war summer; Cfc – Temperate, no dry season, cold; Dsb – Cold, dry summer, warm summer; Dsc – Cold, dry summer, cold summer; Dfb – Cold, no dry season, warm summer; Dfc – Cold, no dry season, cold summer; ET – Polar, tundra.

Fires occurring North of the Arctic Circle are garnering significant attention and are anticipated to increase in frequency in the years ahead^36,37^. The lack of precedents for these fires makes it challenging to predict their behaviour accurately. In Norway, climate change is exacerbating conditions conducive to wildfires, leading to heightened risks and increased occurrences of such events. Rising temperatures and changing precipitation patterns are creating drier conditions, which, combined with longer periods of warm weather, are contributing to the drying out of vegetation and forest fuels. Expected changes in climate will lead to changes in the vegetation due to ecological survival limitations and adaptation to the new expected conditions. According to Köppen-Geiger model Norway will transition to a new paradigm in the coming years and it is expected to have a more fire prone landscape^38^ as Fig. 2 shows. As a result, the fire season is extending, and wildfires are becoming more frequent and severe. Additionally, reduced snowpack and earlier spring thaws are leaving landscapes more vulnerable to ignition. These changes in climate are altering the fire regime in Norway, increasing the likelihood of large-scale wildfires that pose significant threats to ecosystems, biodiversity, property, and human lives. Furthermore, the impacts of wildfires extend beyond the immediate fire zone, affecting air quality, water resources, and long-term ecological resilience. Addressing the challenges posed by wildfires in the context of climate change requires coordinated efforts in fire management, land-use planning, and climate adaptation strategies to mitigate risks and enhance resilience in Norwegian landscapes.

### Fire reports and analysis

To assess wildfire patterns, a national historical record of wildfires in Norway from the Norwegian Directorate for Civil Protection^39^ was utilized. This Norwegian fire occurrence dataset includes the location and date of wildfires spanning from 2016 to the present. It encompasses all fires documented in grasslands, cultivated areas, forests, and uncultivated lands, independently of their ignition source. In this study, we consider the data collected from 1st January 2016 to 21st December 2023, covering 8 years. The total number of fires considered in this study is 9737.

The data are sourced from the fire and rescue service reporting system in Norway, known as BRIS (brann- og redningstjenestens rapporteringssystem)^40^. This dataset includes all fires, not imposing a lower limit on the burned area. However, it is necessary to remark that the data reported in this database, and used in the present study, is not precise regarding the area, being an estimate from the reporting fire officer that determines the size reported. The locations in the dataset represent the GPS locations of the trucks attending the incident. Although these locations may not precisely correspond to the locations where the fire originated, since they correspond to the positions of the first resource to arrive, it is the only reported location that can be associated with these events. The fire reports contain Norwegian fire investigation data compiled since 2016 when DSB initiated the consolidation of data after fires using a uniform nationwide post-fire investigation method. The primary aim of investigating these data reports is to accurately identify the causes, damage, and characteristics of fires and to implement effective fire prevention measures. In terms of this investigation, a fire is defined as a combustion phenomenon that is unintentionally started (by human mishandling, errors, wrong maintenance of electric infrastructures, or naturally mainly by lighting) or caused by arson, requiring the use of fire extinguishing equipment or equally effective means. A single fire is defined as one that has spread from a single point of origin, commencing with fire initiation and continuing until extinguishment. The investigation encompasses all fire phenomena occurring within the territory of Norway. Importantly, the fire reports contain personal and private information. Hence, all fire information presented in this study underwent averaging, summation, and statistical processing. Additionally, any details pertaining to individual cases that were not publicly disclosed were excluded.

To complement the Norwegian fire occurrence dataset, we incorporated satellite imagery analysis to estimate the burned area for each fire incident. Using the latitude, longitude, and date of the fire from the BRIS dataset, burn area was estimated using Landsat 8 Collection 2 Level-2 Surface Reflectance (SR) imagery accessed via Google Earth Engine (GEE)^41^. For each fire event, images were selected within a 30-day window before and after the recorded ignition date, and a 1 km buffer was created around each ignition coordinate to account for location uncertainty and capture localized burn impact. This buffer size was chosen to account for spatial uncertainty in ignition location data, which is common in historical fire datasets, and to capture localized burn dynamics, particularly in low-to-moderate severity fire events. Additionally, it ensured spatial consistency in the burned area calculations across all fire records, providing a standardized approach for analysis. Images were cloud- and snow-masked using the QA_PIXEL bitmask (cloud shadow: bit 3, snow: bit 4, cloud: bit 5), as recommended by the USGS. A mosaic of the filtered images was created for both pre-fire and post-fire periods. The Normalized Burn Ratio (NBR) was calculated by taking the difference between the Near-Infrared (Band 5) and Shortwave Infrared (Band 7) reflectance values, divided by their sum. The difference between pre- and post-fire NBR (dNBR) was used to assess burn severity and estimate burned area. The burned area was classified into severity levels, and the total area affected, particularly in moderate to low severity classes, was calculated in hectares. This method allowed for a more precise estimation of fire-affected areas, compensating for the inherent inaccuracies of the BRIS-reported burned area data. To ensure the accuracy of our total burned area calculations, we implemented checks and adjustments based on conditions during fire incidents, local fire department data, and filtered out satellite imagery affected by cloudy conditions. To address inaccuracies, we excluded cloudy imagery for better satellite analysis, setting burned areas to zero if reported as less than 20 hectares, and adjusting satellite-derived estimates based on fire department data when discrepancies appeared. This method corrected potential exaggerations caused by cloudy conditions or misinterpretations in satellite imagery. We found that 4,512 fires retained their original recorded areas, while 5,225 fires had updated values. This approach significantly enhanced the dataset’s reliability, crucial for effective fire management and response strategies. The data analysis focused on identifying temporal and spatial characteristics of wildfire occurrences across Norway from 2016 to 2023 and evaluating associated climatic drivers. Four key analytical components were undertaken: (1) analysis of temporal patterns to explore trends and seasonal variation in wildfire activity; (2) assessment of spatial patterns of fire distribution by county; (3) computation of a Composite Wildfire Risk (CWR) to classify counties based on frequency and intensity of fire activity; and (4) evaluation of spatiotemporal relationships with climatic variables. The CWR was derived using a multi-criteria decision-making (MCDM) approach that integrated three indicators: number of fires per year (NFPY), average burned area per year (BAPY), and average burned area per fire (BAPF). Each indicator was normalized using min–max scaling, and equal weights (1/3) were assigned to ensure balanced contribution from all factors. The final composite risk score was calculated as the average of the normalized values, and counties were classified into five risk categories using the natural breaks classification method. The classification thresholds used in the analysis are detailed in Table 1.

**Table 1:** Threshold Values for Risk Classification derived using Natural Breaks Classification Method.

Levels	NFPY (fires/year)	BAPY (hectares/year)	BAPF (hectares/fire)	CWR (unitless)
Very low	54	55	0	0.03
Low	66	122	1.0	0.21
Medium	92	177	1.3	0.33
High	152	377	2.0	0.60
Very high	331	379	6.0	0.72

The analysis on wildfire initiation risk in Norway uses specific threshold values to categorize counties based on three main criteria: the number of fires per year (NFPY), the average burned area per year (BAPY), and the average burned area per fire (BAPF). These thresholds help in systematically classifying the risk levels of each county, thereby enabling targeted wildfire management and prevention strategies. The analysis began with extracting data on fire occurrences, including the associated counties and dates over the last eight years. This data was then aggregated and grouped by county and year to calculate the average number of fires per year for each county. Using this aggregated data, the counties were classified into different risk levels based on natural breaks classification, ensuring each class contained an equal number of features. Based on the threshold values for the number of fires per year (NFPY), counties are categorized are mentioned in Table 1.

To merge the three wildfire risk types into a single composite risk classification, a multi-criteria decision-making (MCDM) approach with equal weighting for each criterion was employed. The three risk types considered were the number of fires per year (NFPY), average burned area per year (BAPY), and average burned area per fire (BAPF).

First, the data for each criterion was normalized using the provided threshold values. This ensured that the data from different criteria could be compared on a common scale between 0 and 1. The normalization formula is mentioned in Eq. (1).

1 $$Normalized Value=\frac{Actual Value-Min Value}{Max Value-Min Value}$$

The composite risk score was calculated for each region by multiplying the normalized value of each criterion by its respective weight (1/3 for each criterion), and summing these weighted values. This composite risk score provided an overall measure of the wildfire risk for each region. The composite risk score is calculated by averaging these normalized values in Eq. (2).

2 $$Composite Risk Score=(\frac{1}{3}\times Normalized NFPY)+(\frac{1}{3}\times Normalized BAPY)+(\frac{1}{3}\times Normalized BAPF)$$

Finally, the counties were classified into risk levels based on the composite risk scores. The risk levels were defined using Natural Breaks classification, where the counties were divided into groups based on the distribution of the composite risk scores.

## Results

### Temporal patterns and characteristics of fire occurrences between 2016 and 2023

On average, more than 1,233 wildfires occur per year, burning over 2,036 hectares per year. For comparison, there were 3,175 building fires reported in 2019. Individual wildfires in Norway tended to be small, with an average of 1.64 hectares per fire in the 4594 fires where the burned area is reported. 3756 of these fires reported a burned area of less than 1 hectare. The specific statistics and values largely vary depending on the studied year, as discussed in the following sections.

This section studies the temporal variability of wildfires in Norway by analysing the distribution of events over the years. Both the fire initiation and the fire spread are analysed. Fire initiation focuses on the start of the fire, independently of the cause, which escalates to an uncontrollable scale. It is quantified by the number of fires. Fire spread indicates the extent to which a fire propagates after initiation, and it is measured by the burned area. These parameters largely vary across regions and time, influenced by natural factors such as ignition sources and firefighting efforts.

**Fig. 3:**
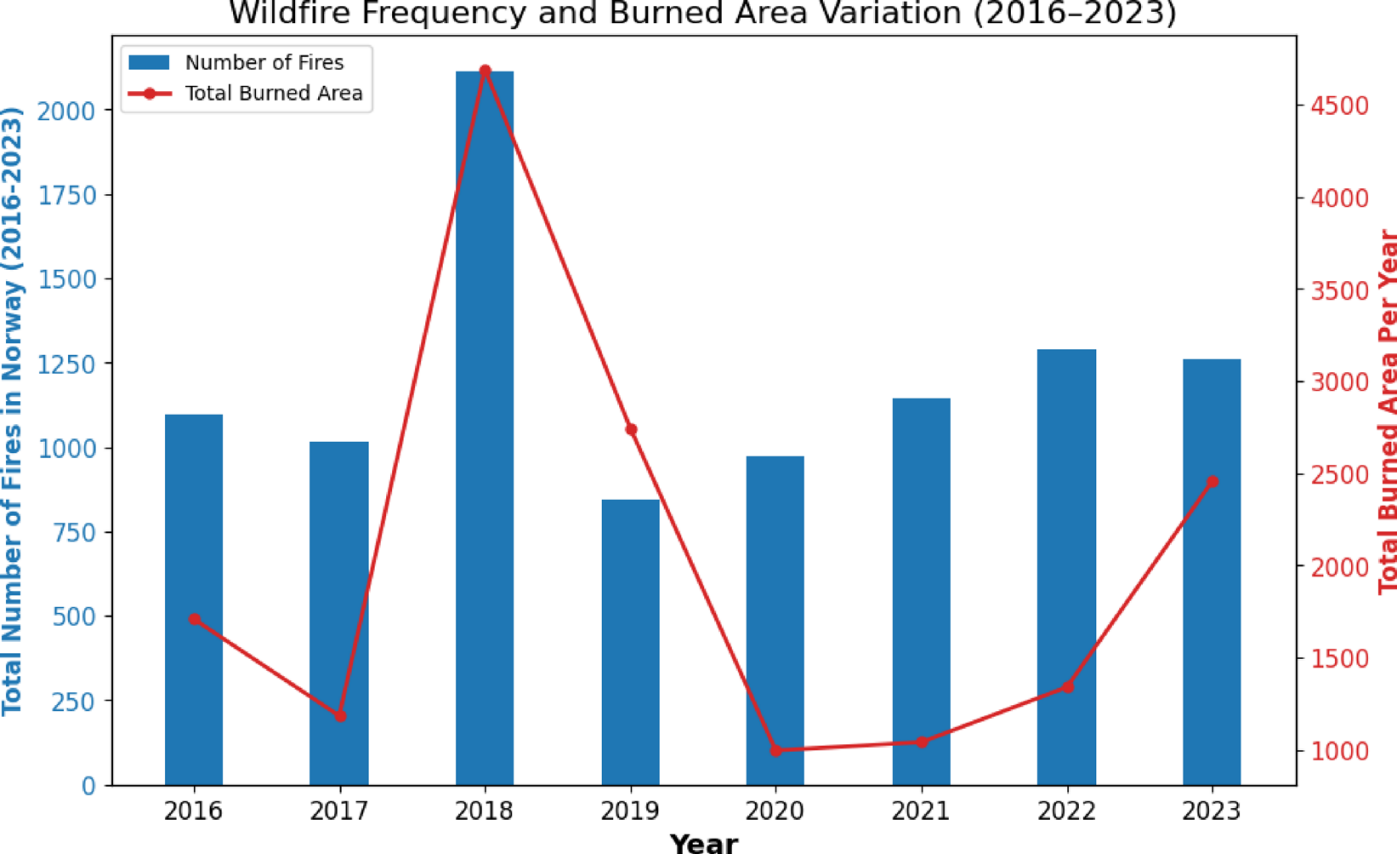
Total number of fires in Norway per year from 2016 to 2024 (blue columns) and total burned area per year (red line).

**Fig. 4:**
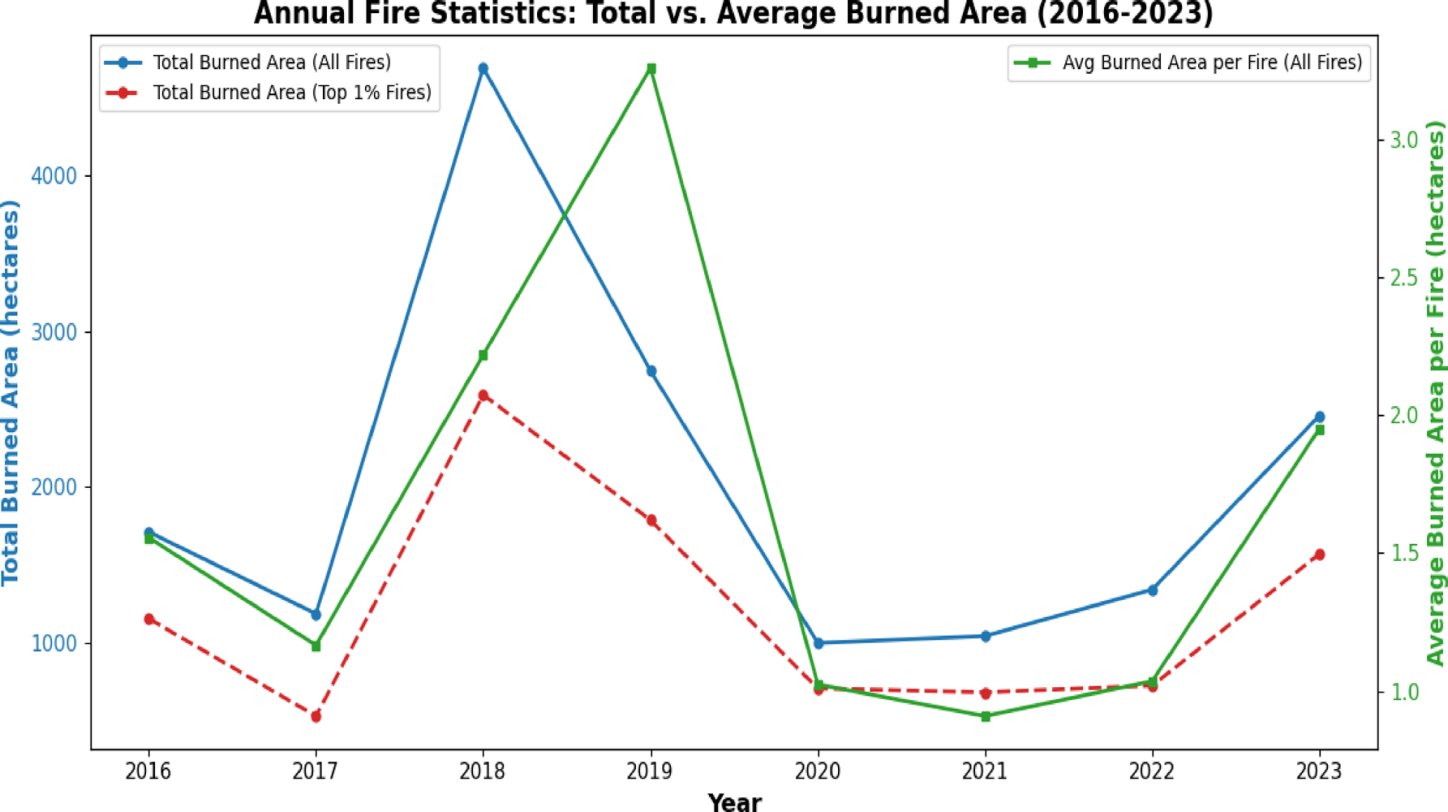
Burned are due to wildfires in Norway in the period 2016–2023. Total burned area (blue) per year taking into account all the fires and the biggest 1% of the fires (dotted red). Average burned are per fire (green) per year taking into consideration all occurred fires.

Complex trends in wildfire activity from 2016 to 2023 are observed from the collected information, as can be observed in Figs. 3 and 4. Initially, from 2016 to 2018, both the number of wildfires and the total burned area experienced notable changes. Specifically, 2018 stands out due to its exceptionally high number of fires (2,115), indicating an intense period of fire initiation with a total burned area of 4691 hectares, suggesting that 2018 experienced severe wildfire events not only in terms of wildfire count but also in size. In contrast, 2019 saw a significant drop in the number of fires (to 842), yet it recorded a total burned area of 2,743 hectares, showing a higher average burned area per fire than 2018. This indicates that while there were fewer fires, at least some of them were probably more extensive. In 2020, a decrease in both metrics was observed: the number of fires was 995, and the total burned area dramatically decreased to 842 hectares, suggesting effective containment and reduced fire spread. From 2021 onwards, the trends show a gradual increase in the total burned area, with it reaching 2,456 hectares in 2023. During this period, the number of fires remained relatively constant, staying above 800 fires per year. This consistent fire count, coupled with the fluctuating burned areas, underscores the complexity of wildfire dynamics. The year 2020 stands out with both the burned area per fire and the burned area for the largest cases exhibiting a decline, indicating that while fire initiation remained significant, the fire spread was limited.

These observations suggest that the fire initiation process has played a more significant role than the fire spread process in the long-term reduction observed. While the number of fires has remained high, the ability of these fires to spread and cause large-scale damage has varied, highlighting the importance of addressing both fire initiation and spread processes to manage and mitigate wildfire impacts effectively. Alternatively, the extent of burned areas per fire showed notable yearly fluctuations, and the pattern of these changes was similar for both the largest fires and the overall total burned area.

**Fig. 5:**
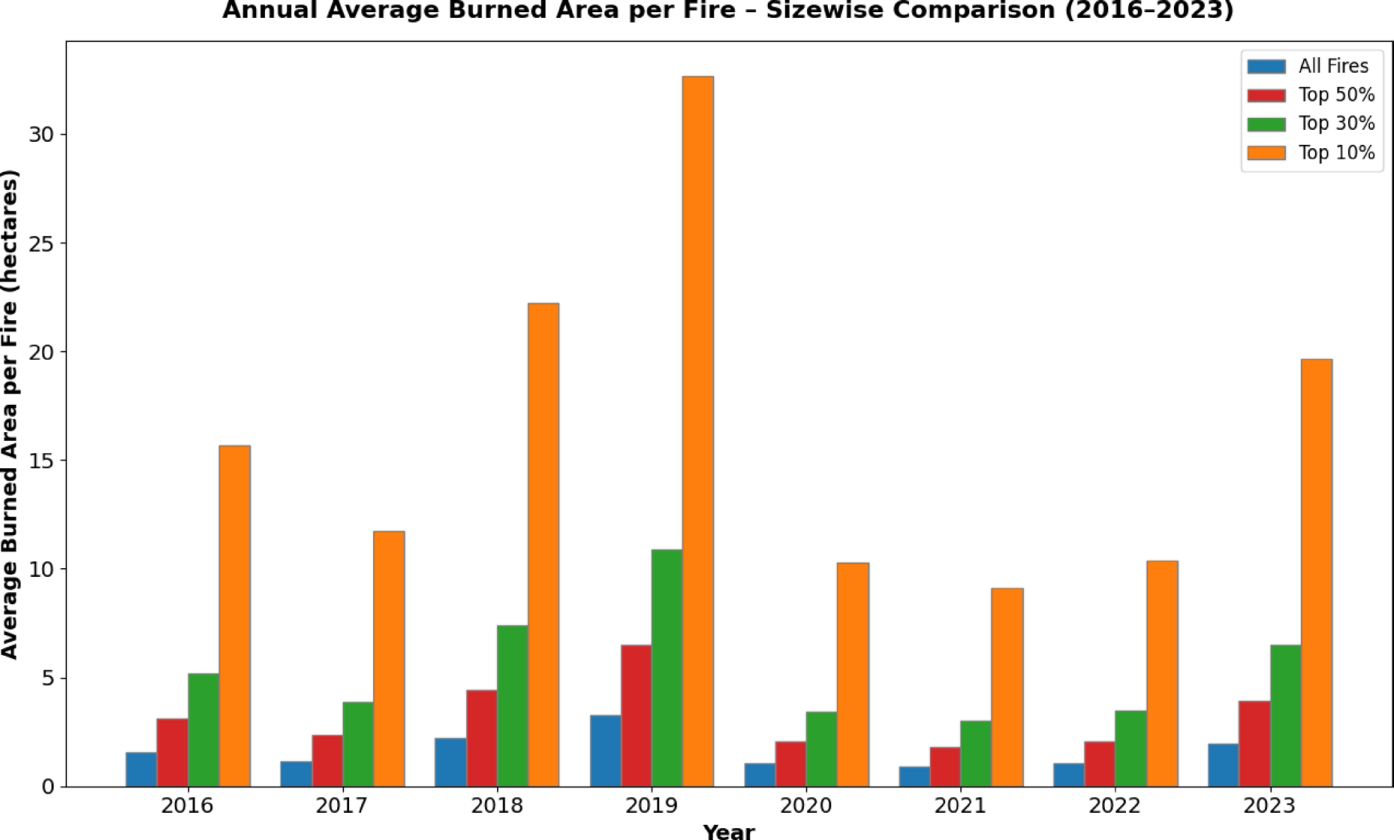
Annual burned area per fire in the period 2016–2023 analysing all the fires (blue), top 50% of the fires (red), top 30% of the fires (green), and top 10% of the fires (orange). To the left, burned are and to right average burned area.

This means that the way the size of the biggest fires varied each year matched the overall yearly changes in the total area burned. Additionally, Fig. 5 represents the annual average burned area per fire categorized by size percentiles (100%, top 50%, top 30%, and top 10%) from 2016 to 2023, highlighting the disproportionate impact of the largest fires on the total burned area. In Fig. 5, the combined burned area of the top 10, 30, 50, and all largest fires each year is shown. The results demonstrate a clear disparity in the burned area contribution between large and small fires. The top 10% of fires, categorized by size, consistently account for the majority of the burned area each year, with a notable peak in 2018 and 2019, reflecting the occurrence of exceptionally large fire events during these years. In contrast, the average burned area for all fires (100% category) remains relatively low, suggesting that the majority of fires are small and have limited impact. This highlights the disproportionate influence of large fires on overall fire damage and underscores the importance of targeted management strategies aimed at preventing and mitigating large fire events. Consequently, the total burned area each year is mainly influenced by a few very large fires rather than smaller fires. This means that a small number of big fires have a much greater impact on the overall burned area than many smaller fires combined. Thus, understanding the factors influencing large-scale wildfires is crucial when analyzing yearly variations in burned areas. Given that our study period only spans 8 years, from 2016 to 2023, the impact of climate change is difficult to detect. However, the increase in the number of events in 2018 is However, it is important to note that the annual variation in the burned area per fire is influenced by large-scale events, underscoring the importance of understanding natural factors such as extreme dryness and strong winds. These natural factors can play a significant role in determining the severity and extent of wildfires, alongside social changes.

**Fig. 6:**
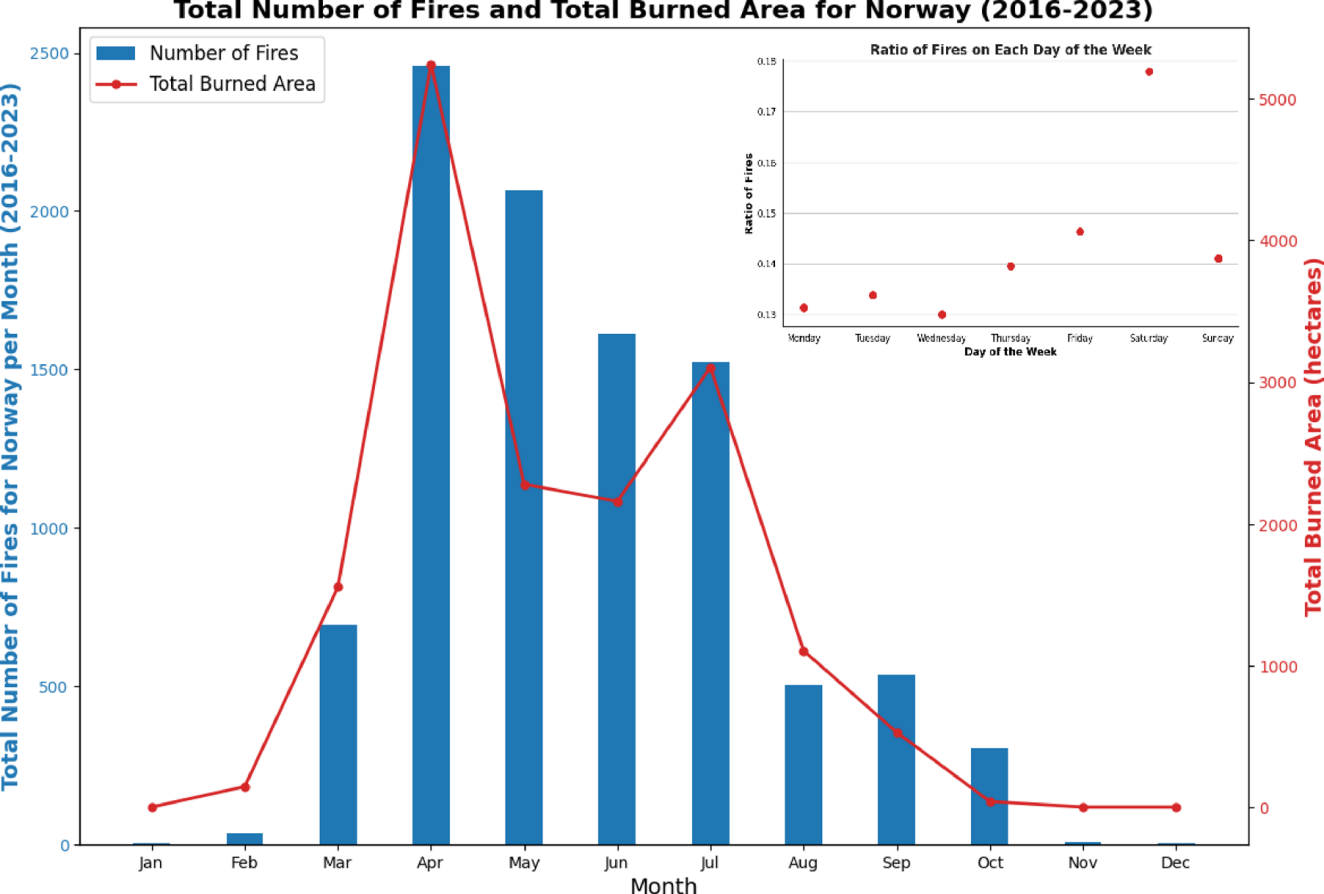
Total number of fires and area burned per month in the period 2016–2023 in Norway. On the top right, ratio of fires depending on the day of the week during the period 2016–2023.

It has also been observed a shift in Norway’s fire season from the conventional summer months to a more predominant occurrence during spring, particularly from April to July (Fig. 6), which can be attributed to several interconnected factors. As winter transitions to spring, the protective snow and ice cover on the landscape gradually diminish, leaving vegetation exposed. This exposure allows the vegetation to dry out rapidly due to increased sunlight and temperatures characteristic of the spring season. By April, when the protective winter shell has dissipated significantly, the vegetation reaches a critical point of dryness, rendering it highly susceptible to ignition. Additionally, the weather patterns in April often feature lower precipitation, which further exacerbate the dryness of the vegetation and increase the risk of fires igniting and spreading rapidly. This combination of factors creates a perfect storm for wildfires, resulting in a clear maximum in the number of fires and the total area burned during April. The heightened dryness of the vegetation, coupled with conducive weather conditions, culminates in a peak fire occurrence during this month^42^.

Additionally, seasonal changes are also influenced by anthropogenic conditions, such as an increased number of visitors for recreational purposes. A high number of fires in April and early May starts around fishing or camping sites (fireplaces mishandled) and around areas of great affluence of people (big cities). Figure 6 illustrates another temporal characteristic, depicting the number of fires and the area burned by the day of the week. It is worth noting that fires are more frequent on weekends than weekdays, hinting at a link with recreational pursuits. Notably, Fridays and Saturdays stand out with a surge in fire incidents, suggesting heightened outdoor activities during these periods. Moreover, the number of fires tends to be higher during weekends in March, April, and May. In the seasonal variation observed in Fig. 6, the substantial disparity between the highest and lowest data points ranging from 5 to 10 times indicates a pronounced influence of natural conditions. This suggests that seasonal changes significantly impact the behaviour of fires. Notably, while fire initiation is predominantly influenced by seasonal variations in natural factors, such as temperature and humidity, it’s essential to recognize that both fire initiation and fire spread are subject to seasonal effects. This means that not only does the likelihood of fires starting change with the seasons, but also the extent to which they propagate across landscapes. For instance, during dry and hot seasons, the conditions favour both the ignition and rapid spread of fires, whereas during cooler and wetter periods, fire occurrence and propagation may be limited. Thus, understanding the seasonal dynamics is crucial for assessing fire risk and management strategies.

### Spatial patterns of wildfire occurrences

**Fig. 7:**
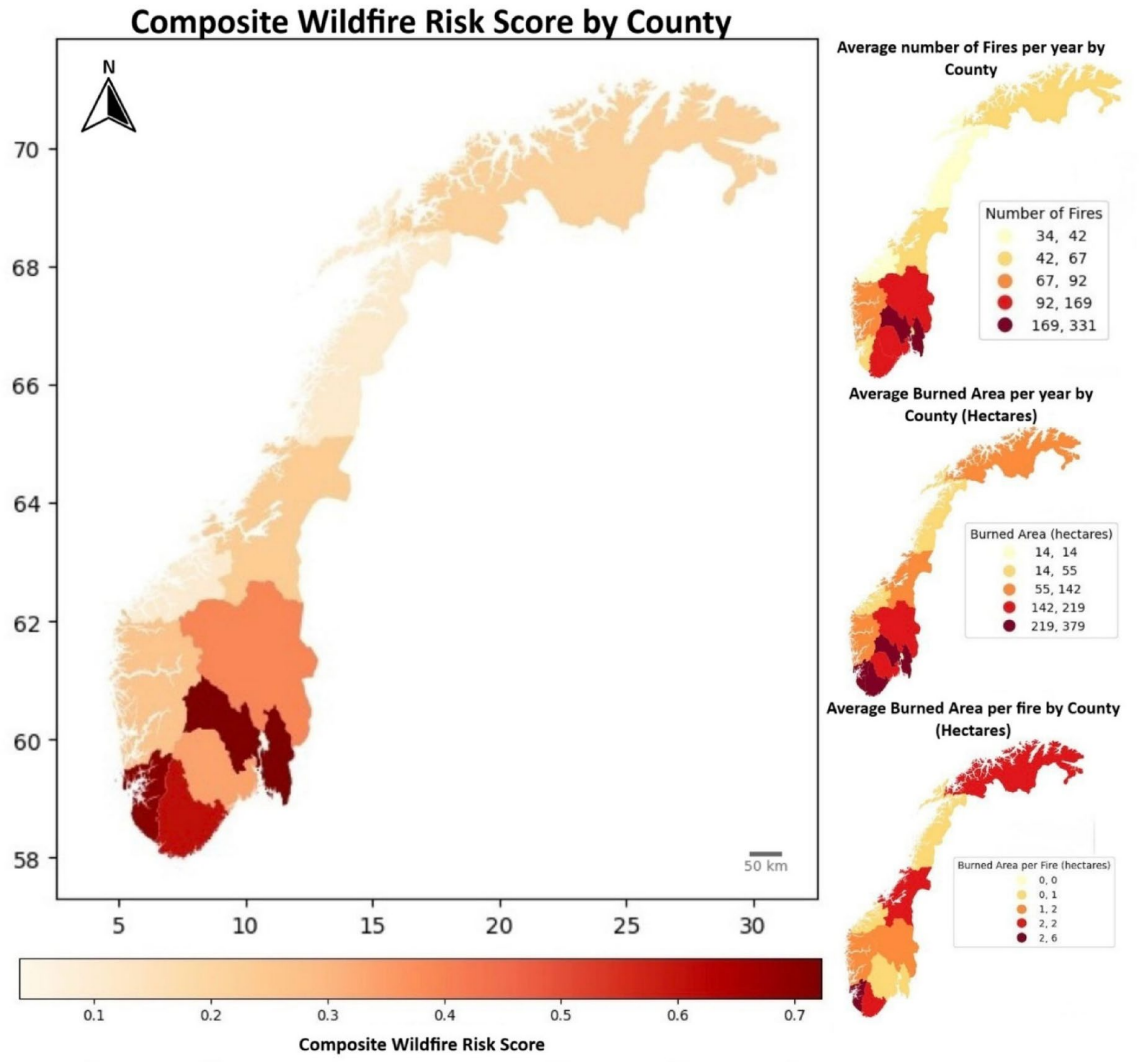
Composite wildfire risk map of Norway by county shown in the left part. Right part, from top to bottom, shows the number of fires, the burned area, and the burned area per year in each county during the period 2016–2023. The maps were generated using Geopandas (v0.10.2, https://geopandas.org/), Matplotlib (v3.4.3, https://matplotlib.org/), Seaborn (v0.11.2, https://seaborn.pydata.org/), and Mapclassify (v2.4.3, https://pysal.org/notebooks/explore/mapclassify/intro.html) in Python.

The location of wildfires in the last years also gives an important information regarding the expected future occurrence of events. The analysis of the average number of fires and area burned in the last 8 years is represented in Fig. 7, where the spatial variation of wildfire occurrences by county is shown, being evident that the number of fires tends to be higher in the southern regions, particularly in the southeast. Southeast is comprises of counties such as Innlandet, Viken, Oslo, Vestfold and Adger. In southeast where some counties experienced over 100 wildfires per year. Compared to that, the Southwest—which comprises counties like Rogaland and Vestland—experienced fewer wildfires. As explained before, Southeast is characterized by drier conditions and denser population in counties like Viken and Oslo compared to the northern other regions, characteristics known to contribute to the occurrence of wildfires. Conversely, the northern part of Norway (Nordland, Troms og Finnmark) shows fewer wildfire cases, totalling approximately 450 on average approximately 96 fires per year cases over 8 years. Figure 7 displays the spatial distribution of the burned area, which exhibits a similar distribution except for Vestfold and. Counties such as Rogaland, located in the western coast, recorded the highest values of burned areas. In comparison, and despite the high number of fires in the Viken Vestfold region, the burned area there was relatively smaller than Rogaland.

Figure 7 also indicates the burned area per fire, which was notably high in Rogaland and Agder. To better highlight spatial variation, the national average rate was determined. It was observed that the number of fires was higher in the Eastern areas, which are the most densely populated regions in Norway and experience dry conditions in winter. In this study, the fire season was defined as the period encompassing consecutive months with a number of wildfires greater than 100. The fire season typically commences around early March in the southern part of Norway and gradually progresses northward over approximately three months, beginning around late May in Finnmark. The shift in the wildfire season is likely influenced by factors such as dryness and vegetation patterns. Moreover, heavy snowfall in the northern part of Norway during winter months may explain the smaller number of wildfires compared to other seasons, as snow-covered surfaces inhibit fire occurrence. Regions in central Norway exhibit unique characteristics, where the wildfire season starts slightly later and lasts longer. This could be attributed to the region’s specific weather patterns and population density. Additionally, since hydrological factors contributing to wildfire occurrence vary across regions, it is challenging to determine the dominant factors for wildfire occurrence. Future research should focus on identifying regional and seasonal drying factors and their spatial characteristics to better understand wildfire occurrences.

### Composite wildfire risk (CWR)

The composite wildfire risk (CWR) classification reveals significant disparities among the counties, particularly highlighting those with very high and very low risk levels. Counties such as Rogaland, and Viken have been categorized as “Very High” risk areas. This classification indicates an exceptionally high frequency and intensity of wildfires in these regions, necessitating urgent attention and substantial resource allocation for wildfire prevention and management. (see Fig. 7) The very high composite risk scores for these counties suggest that they consistently experience large numbers of fires, significant burned areas per year, and considerable burned areas per fire, emphasizing the need for proactive and robust wildfire mitigation strategies.

On the other end of the spectrum, More og Romsdal and Nordland are classified as a “Very Low” risk area, with a composite risk score indicating minimal wildfire activity. This low-risk classification implies that these counties experience a significantly lower frequency and intensity of wildfires compared to other regions. As a result, fewer resources may be required for wildfire management in these regions, allowing policymakers to focus efforts on higher-risk areas. The distinct contrast between these counties underscores the importance of tailored wildfire management strategies that account for the specific risk levels of each region. By prioritizing interventions in very high-risk areas and maintaining vigilance in very low-risk areas, stakeholders can effectively mitigate the impact of wildfires across Norway.

The spatial distribution of wildfire risk across Norway, as revealed by this analysis, provides critical insights for wildfire management strategies. High and very high-risk counties require more focused attention and resources for wildfire prevention and management. Understanding the areas with high average burned areas per fire can help in developing targeted mitigation efforts to reduce the impact of each fire incident. Implementing proactive measures in counties with higher fire risks can help mitigate the impact on communities and ecosystems. In conclusion, the systematic approach utilized in this analysis offers valuable insights into the spatial distribution of fire risk across Norway. By categorizing counties based on different aspects of fire activity, policymakers and stakeholders can prioritize interventions and allocate resources more effectively to reduce the impact of wildfires. This analysis underscores the importance of comprehensive wildfire management and prevention strategies tailored to the specific risks of each region.

### Spatial temporal characteristic of wildfire with respect to climatic factors

^2^Understanding climatic factors is crucial for assessing wildfire risk, particularly in diverse geographical regions like Norway. Wildfires can have significant impacts on ecosystems, biodiversity, and human livelihoods. The occurrence and severity of wildfires are closely linked to climatic conditions, especially temperature, wind speed, and precipitation. Higher temperatures and drier conditions generally increase the likelihood and intensity of wildfires ^43,44^. The climatic variables used in the correlation analysis, wind speed, precipitation rate, land surface temperature (LST), and Palmer Drought Severity Index (PDSI), were obtained from globally recognised remote sensing and reanalysis datasets to ensure accuracy and consistency across Norway^43–45^. Wind speed and precipitation data were sourced from the ERA5 reanalysis dataset provided by the European Centre for Medium-Range Weather Forecasts (ECMWF)^43^, with 10-m U and V wind components processed into scalar wind speed (m/s), and total precipitation (mm/day), including rainfall and snowfall. Both variables were originally available at an hourly temporal resolution and a spatial resolution of approximately 0.25° × 0.25° (31 km), and were aggregated to monthly averages and sums, respectively, for the period 2016 to 2023. Land surface temperature (LST) was derived from MODIS Terra (MOD11C3) and Aqua (MYD11C3) Level-3 products^44^, offering monthly data at 0.05° (5.6 km) resolution. The Palmer Drought Severity Index (PDSI), reflecting long-term dryness patterns, was obtained from the TerraClimate dataset developed by the University of Idaho^45^, with monthly data at 0.0417° (4 km) resolution for the same period. All datasets were clipped to the national extent of Norway, and monthly spatial averages were calculated to ensure temporal alignment and consistency across the climatic indicators. This preprocessing ensured that the climatic indicators aligned temporally with monthly fire count records, enabling a consistent basis for the correlation analysis.

**Fig. 8:**
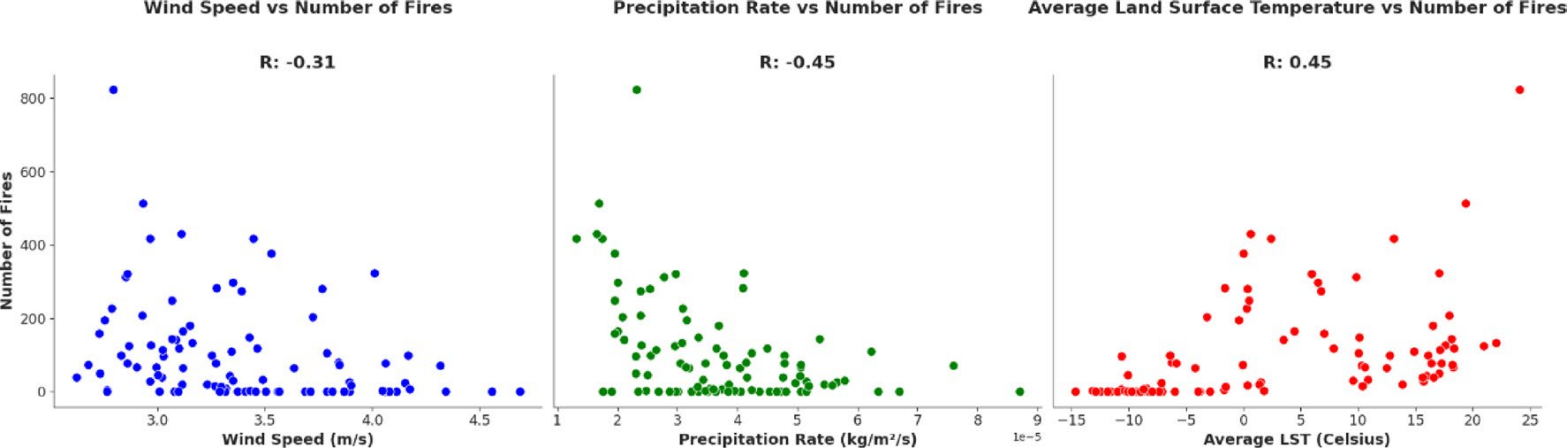
Relation between climatic factors (wind speed, precipitation and Land Surface Temperature) and number of fire in Norway on a monthly basis in the period 2016–2023.

As previous studies^14,46,47^ pointed out, both the absence of rain and the amount of rainfall are essential in determining fire risk. Higher temperatures and stronger winds typically increase the risk of fires. Interestingly, Grimsrud found that "average wind speed for the month" surprisingly decreases fire risk, possibly because larger storm systems with high winds often bring moist air, reducing fire danger. In this study, we investigated the relationship between wildfire activity and three key climatic variables: land surface temperature (LST), precipitation, and wind speed. These were selected based on their relevance in previous literature and availability from remote sensing datasets. We calculated Pearson correlation coefficients between the monthly average values of these climatic variables and the monthly fire counts across Norway for the period 2016–2023. This allowed us to quantify the linear relationship between each factor and the number of fires. By using a scatter plot to illustrate the relationship, we derived several insights (See Fig. 8). Our findings show a negative correlation between the number of fires and both wind speed (− 0.31) and precipitation rate (− 0.45). On the other hand, there is a positive correlation (0.45) between the number of fires and average LST. This suggests that higher temperatures increase fire likelihood, whereas higher wind and precipitation reduce it. These results are consistent with previous observations, including Grimsrud’s^14^ interpretation of wind and moisture dynamics.

**Fig. 9:**
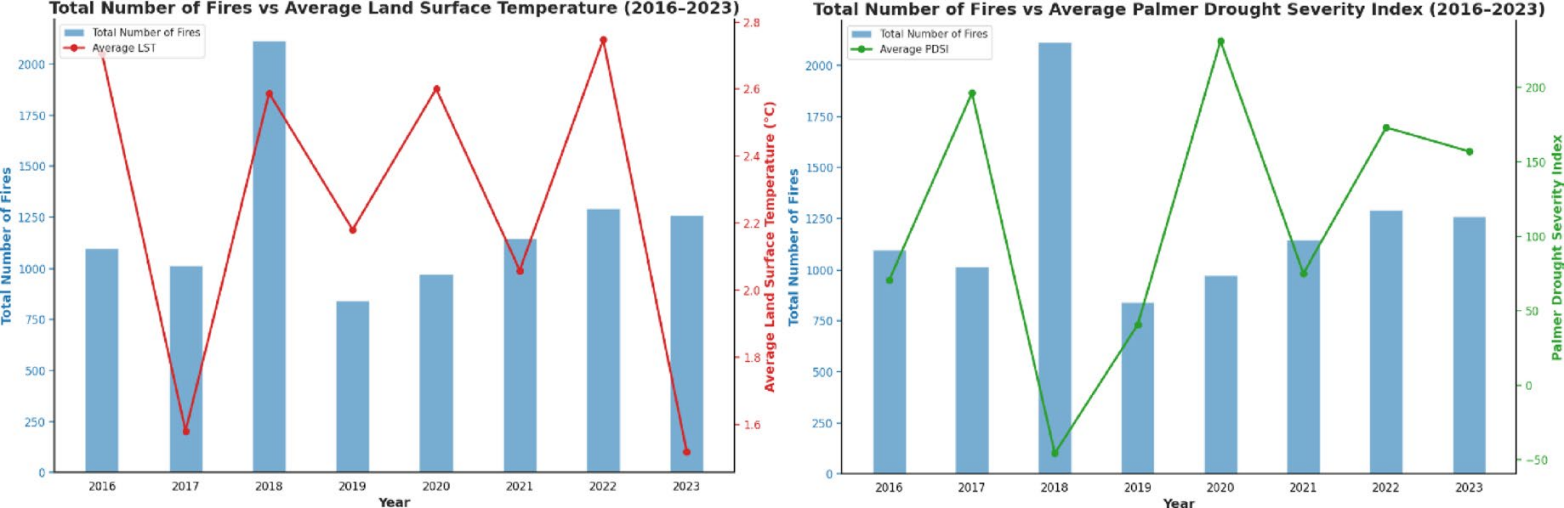
Number of Fires vs Palmer Drought Severity Index and Land Surface Temperature (2016–2023).

To assess the potential role of longer-term dry conditions, we also examined the Palmer Drought Severity Index (PDSI)^44^. The PDSI is a well-established drought indicator that captures extended dryness patterns. Our comparison between annual PDSI values and fire counts revealed that 2018, a year with extreme drought and unusually high LST, recorded the highest number of wildfires. This reinforces the link between prolonged dry spells and fire outbreaks. This year was also characterized by very high land surface temperatures, as our annual analysis of LST against wildfire count showed a similar pattern as presented in Fig. 9.

It’s important to note that our correlation analysis does not imply causality, but rather highlights statistical relationships. We acknowledge the limitations of using correlation alone and the importance of considering both current and antecedent climatic conditions. While this preliminary analysis is useful for identifying trends, future work will include time-lagged correlation and multivariate regression models to more accurately quantify the influence of pre-conditions on wildfire ignition and spread.

By leveraging remote sensing data such as land surface temperature , precipitation records, wind datasets, and PDSI imagery in conjunction with wildfire occurrence data, this study provides a foundation for understanding the spatiotemporal dynamics of wildfires in Norway. These insights are valuable for developing early warning systems and proactive mitigation strategies.

## Current management strategies

### Fire regulations

In Norway, there are no specific regulations related to wildfire management, control or protection. In this section, the three regulations that rule fires are described, and the mention on wildfires that they include, if so. These three documents are: the Act on protection against fire, explosion and accidents involving dangerous substances and on the fire service’s rescue tasks (LOV-2003-06-14-20)^48^; the Regulation on fire prevention (FOR-2015-12-17-1710)^49^; and the Regulations on the organisation, staffing and equipment of the fire and rescue services and the emergency reporting centres (FOR-2021-09-15-2755)^50^.

The purpose of the Act is to protect life, health, the environment and material assets against fire and explosion, against accidents involving dangerous substances and dangerous goods and other acute accidents, as well as unwanted intentional incidents.

The Act is superior and sets general requirements for the public and municipalities in prevention, and preparedness. It establishes that everyone has to act in a proper manner to prevent fires. If fires occur, it must be immediately reported to the emergency response centre. If possible, the effects of the damage should be limited (ref. § 5). Additionally, every owner of a building, vehicle, area (forest), must take necessary measures to prevent and limit fires. Additionally, the owner must establish surveillance after a wildfire (ref. § 6). It also states that the municipality is responsible for establishing a fire service for handling local risks in the municipality, both in terms of preventive and preparedness measures (ref. § 9). In addition to firefighting, the fire department must conduct informational campaigns for the public, property owners, and others, as well as engage in preventive measures to avoid all kind of fires (ref. § 11). The municipality has the opportunity to establish extraordinary measures when necessary. For example, during high wildfire danger, a total ban on open fires may be imposed (ref. § 14). Have any reference to wildfires.

The Regulation on fire prevention should help reduce the likelihood of fire, and limit the consequences that fire can have on life, health, the environment, and material values. It supplements the requirements set by the Act at a more detailed level. In this regulation, the prohibition to use fires or handle flammable objects in or near forests and other uncultivated land from April 15th to September 15th is established (ref. § 3). It also defines that the municipality should conduct fire prevention work based on assessed risks (ref. § 14–16).

The Regulations on the organisation, staffing and equipment of the fire and rescue services and the emergency reporting centres aim to a. set requirements for the municipalities regarding the organisation, staffing and equipment of fire and rescue services and emergency reporting centres, b. sets requirements for the municipalities regarding the competence of personnel in the fire and rescue services and emergency reporting centres must have c. shall contribute to reducing the probability and consequences of fires and other accidents. On this, these regulations establish that the municipality is responsible for establishing a fire service based on a risk analysis, emergency preparedness analysis, and preventive analysis. There are some requirements that must be met, and then the fire service have to be adapted to local risks (ref. § 6–9). It also defines that reinforcement resources have to be established in municipalities/regions where there can be a significant risk for wildfires (ref. § 16). There are no specific equipment requirements, but three must be equipment to handle incidents they are designed for based on their analysis (ref. § 19). This regulation is not focused on wildfires, but mentions it in paragraph 16, where it establishes the reserve forces for wildfires and other incidents, saying that *If the fire and rescue service’s own emergency forces do not have sufficient personnel resources to handle emergency situations that may occur, including in areas where there is a significant risk of wildfire, the fire and rescue service alone or in cooperation with other fire and rescue services must ensure have sufficient reserve forces for wildfires and other incidents. The need for reserve forces must be evident from the preparedness analysis.* It also clarifies that *the reserve force must be trained for the tasks*.

Finally, it is important to notice that fire prevention can also be conducted through maintenance of the landscape, not only fire regulations. Poorly managed landscapes can be highly flammable and the cost of incidents happening has been proven to be much higher than the costs of covering preventive measures. There is no existing previous data to prove this point, but several analysis has shown that increased management of the landscape reduces the risk of fire considerably, mainly in periods when weather conditions increase this risk. In areas where vegetation is highly flammable, such as the heathlands in coastal Norway, a management plan is the most important management measure^14^, ensuring a reduction in the number of fires and the related costs.

### Operational strategies

The Ministry of Justice and Public Service has the overall responsibility for fire protection in Norway. The Directorate for Civil Protection (DSB), on behalf of the Ministry of Justice and Public Service, is the national fire authority and the overall task is to maintain a complete overview of various risks and vulnerability in general. DSB has a role that means that the directorate has subject, administrative and supervisory responsibilities, but does not have the authority to issue instructions to the municipalities’ fire and rescue services^51^. Fire services’ responsibilities cover local, regional and national preparedness and emergency planning, fire safety, electrical safety, handling and transport of hazardous substances, as well as product and consumer safety^52^.

Fighting the fires is the municipalities’ responsibility through local fire and rescue services. Municipalities are responsible for establishing and operating a fire and rescue service, whose main tasks include conducting fire prevention activities and serving as an intervention force in the event of a fire. The fire and rescue service should also serve as an intervention force in other incidents as determined based on the municipalities’ risk and vulnerability analysis.

As of February 2024, there were 197 fire and rescue services in Norway, employing approximately 12,000 employees, including 4200 full-tome and 7800 part time staff^52^. With 357 municipalities in Norway, there are several collaborations between them, resulting in fewer fire departments than municipalities. In order to support smaller municipalities, or any fire station during an event that cannot be controlled exclusively by the nearest fire station, collaboration between fire stations in different municipalities are essential. It is up to each municipality to decide how extensive the collaboration will be. When it is necessary, an emergency alert goes to one of the 12 112-centers in the country.

There are 609 fire stations distributed in 385,207 square kilometres country^52^. The placement of fire stations is based on response time requirements to populate areas and/or buildings with specific risks, with response times requirements typically set to 10 to 20 min. From the total number of fire stations in Norway, 68 of them reported to have special competences for wildfires fighting in 2022. Many municipalities have established an extra wildfire reserve. Usually, the fire service is in charge when wildfires occur, but if lives or health are threatened, the police have the role of the overall incident commander, and other commanders integrate into a joint leadership structure. In the wildfire services planning framework, other resources may also be relevant. The forestry industry can contribute to extinguish and during post-extinguishment. Local farmers contribute to the efforts too, as well as volunteers. This working procedure is known as the Telemark model^53^. Table 2 shows how the emergency preparedness cooperation complements each other with materials and tasks, in connection with wildfire drills and incidents.

**Table 2:** Telemark model working procedure for collaboration on the fight of wildfires in Norway^53^.

Civil defence	Fire department	Wildfire troopers	Forest industry
4’’ hose	2 ½ ‘’ hose	1 ½ ‘’ hose	
2 ½ ‘’ hose	1 ½ ‘’ hose	1 ‘’ hose	
Heavy water supply	Water supply	Light water supply	
Large pumps	Medium pumps	Light weigh pumps / Self-erecting water tubs	Forestry machinery, load carriers, forestry packages
Own crews	Firefighters	Own crews	Employees, machinery
Water supply, KO operation, transport	Offensive extinguishment	Defensive extinguishment	Transport, logging streets

Collaboration is essential in handling wildfires, and incidents are coordinated by the fire department’s 110 emergency call centre. There are 12 such centres in Norway, to which all fire services are obligated to be connected^52^.

Since 2008, there have been several major events in Norway. On June 9, 2008, a significant wildfire started in Froland municipality in Aust-Agder, where several cabins and 1900 hectares of productive forest were lost. The fire was very resource-intensive, with up to 16 helicopters and between 250 and 300 personnel involved in the firefighting effort. In January 2014, during eleven days two very large wildfires took place simultaneously. In Flatanger (Nord-Trøndelag), a grass fire resulted in the loss of 64 buildings, including 23 residential and recreational houses. In Fræoya (Sør-Trødndelag), a fire started in heather in an area of 10 square kilometres, resulting in the loss a residential house. Extensive-evacuations were required in both incidents.

2018 is so far the record year for forest and nature fires. Thorough 2018, more than 2 000 fires in grass and forest were recorded due to an extremely dry summer in southern and eastern Norway. Nearly 1 000 of these fires were wildfires. Much of the emergency response apparatus in the affected areas was mobilized. Calm wind conditions helped prevent the situation from spiralling out of control. All these events have formed the basis for the wildfire regime we have today, including competency requirements, local, national and international cooperation, information campaigns, research and more.

In a report published by DSB in 2019^51^, they recognised the existing challenges on wildfire fighting as: competence, organisation, responsibility, and authority. In a local level, the list of challenges included personnel resources, competence, material, local management and organisation, communication during extinguishing work, and relationship with other actors. In regional coordination, they focused on coordination of the fire service’s resources, coordination of the emergency services efforts, and managing social consequences. They highlighted the limited number of fire fighters with wildfire expertise. In the years from the publication of this report (the last one available), several efforts have been made to improve the situation.

One of the main improvements is the availability of helicopters to combat these situations. To this end, DSB started a collaboration with a private helicopter company through which there is at least one available helicopter for wildfire fighting from April 15th to August 15th. One helicopter is always available, and the possibility of having others is open. As an example, in 2018 there were 22 helicopters available at the same time. As a comment, Norway has good access to light helicopters, but since 2018 there has been very limited access to larger helicopters.

Linked to the use of helicopters, DSB has coordinated a national management support scheme in which Rogaland brann og redning IKS points out fire commanders from several fire and rescue services with expertise on wildfire management. It is a requisite that all the commanders have done bushfire training and exercises and are well equipped to handle the management of bushfire fighting. One leading commander supports the fire chief who is responsible for a wildfire. The responsibility of managing the wildfire will always lie with the fire and rescue service that has the fire in their area of responsibility^14,46,47^.

## Recommendations

As a main recommendation, the different entities involved in wildfire management must understand the inevitable increase in dangerous situations that will come soon. It is important to study the most probable location of these fires, which can be derived from the composite wildfire risk developed in this work. The location, as well as the most probable time (of the year and of the week) when these events will occur, have been described and need to be taken into account for the development of preventive activities to avoid these events from spreading.

In a more particular focus, the Norwegian Directorate for Civil Protection (DSB)^3^ made a number of recommendations that can be highlighted as follows:

- Ensure adequate wildfire helicopter readiness with mandatory management support for extinguishing wildfires.
- Consider how to facilitate the establishment of voluntary cooperation agreements between fire and rescue services and forestry contractors in wildfire preparedness.
- Work towards effective wildfire monitoring and examine how the scheme can best be continued.
- Clarify how wildfires and natural events are to be handled in protected areas.
- Facilitate Norway’s active use of new technology and collaborate with other countries on the development of systems that both prevent and manage fires.
- Contribute to the use of new technology that can enhance fire prevention and extinguishing, including through the use of artificial intelligence (AI).

Additionally, by analysing the data and the current situation in Norway regarding wildfire handling, several points should be addressed to ensure complete and correct treatment of these incidents:

- Implement ways to systematically analyse the territory in every Fire and Rescue Service (F&R) in a homogenized way to be able to compare at a national level.
- Improve and increase the number and quality of data registered in the database (ignition point, local weather data, accurate perimeter, fuel type, etc.)
- Implement a double-check system in the register of data in the database to ensure that correct numbers of burned area are inserted.
- Upgrade and standardize knowledge and training of F&R fire and rescue at all levels (basic firefighters, brigade leaders, incident commander and administrative staff) with the aim of increasing situational awareness, performance and basically safety. There are international standards that could be implemented and followed in Norway.
- Implement levels and categories of the emergency to establish priority rankings and operational phases to reassign resources if needed in a given situation.
- Implement or improve the collaboration between F&R, DSB and Academia from a problem–solution perspective.
- Define the WUI problem in Norway due to the huge vulnerability of its infrastructures and start to investigate and implement planning and prevention measurements to avoid scaling the emergency in a given moment.
- Determine levels of vulnerability of the landscape/ territory and define actions to reduce the potential risk associated to that vulnerability.
- Complex and large events need to be addressed in a knowledge-based manner, both preventively and in preparedness, and a capable organisation is necessary to this end.

## Conclusions

In conclusion, our composite analysis of wildfire data from 2016 to 2023 reveals critical insights for improving wildfire management and preparedness in Norway. The conditions in 2018, which required extensive emergency response efforts, demonstrate the need to strengthen current strategies. Although calmer wind conditions helped limit further spread, still the event highlights the importance of better planning and prevention measures to address future wildfire risks. Consequently, there have been notable improvements in competence requirements, cooperation frameworks, information campaigns, and research initiatives.

Our findings highlight that higher temperatures and drier conditions generally increase both the likelihood and intensity of wildfires in Norway. A positive correlation with land surface temperature (r = 0.45), and negative correlations with wind speed (r = − 0.31) and precipitation rate (r = − 0.45), support previous research, such as Grimsrud (2018). The Palmer Drought Severity Index (PDSI) further confirms that extended dry periods, like in 2018, are strongly linked to increased fire activity. Although the 2016–2023 dataset is relatively limited, the trends indicate a growing need to prepare for longer fire seasons and expanded risk zones under changing climate conditions.

Spatial analysis identified southern counties such as Rogaland and Viken as “Very High” risk areas, requiring urgent attention and greater resource allocation, while regions like Møre og Romsdal and Nordland were found to be at “Very Low” risk. Seasonal patterns show wildfire activity peaking in April and May, especially near recreational and urban areas, emphasizing the importance of fire safety measures in these zones. Fires are more frequent on weekends, linked to increased human activity, while larger burned areas on weekdays suggest potential delays in detection or suppression. The earlier fire season onset in southern Norway, compared to the north, reflects climatic differences. Monitoring LST and related indicators can support proactive and region-specific wildfire management strategies.

In response to the wildfire management challenges identified by the DSB (2019), including issues related to competence, coordination, and resource allocation, several targeted measures have been implemented. These include enhanced helicopter availability during the fire season (April 15–August 15) and the establishment of a national support scheme led by Rogaland brann og redning IKS, deploying experienced fire commanders to assist regional chiefs. To further strengthen preparedness, a shift toward area-specific strategies is essential, particularly for high-risk counties like Rogaland and Viken. Strengthening cooperation between fire services and forestry contractors, maintaining helicopter readiness, and integrating technologies such as AI and satellite monitoring are critical next steps.

Our findings support DSB’s recommendations, emphasizing systematic territorial analysis, improved data quality, and standardized training for fire and rescue personnel. Moreover, fostering stronger collaboration among fire services, DSB, and academic institutions will help develop more robust wildfire strategies. Addressing Wildland-Urban Interface (WUI) risks, enhancing post-fire landscape restoration, and regularly updating wildfire risk maps remain key to building long-term resilience and ensuring effective wildfire preparedness across Norway.

The regulations and operational forces are not fully prepared for the emerging conditions anticipated with future wildfire scenarios. However, by following several targeted suggestions, the situation can be significantly improved. Implementing proactive measures, informed by past events and comprehensive current data, will enhance Norway’s capability to manage and mitigate wildfire risks. This integrated strategy will not only reduce the adverse impacts of wildfires but foster resilience and adaptability in the face of changing climatic conditions. Even with the inherent uncertainties, the certainty remains that readiness and preventive actions are imperative. These efforts will ensure a safer environment, where preventive measures and protective actions are effectively designed and implemented when necessary, thereby protecting lives, property, and the environment.

## Data Availability

The dataset analysed during the current study is the BRIS dataset that can be accessed at www.brannstatistikk.no
